# National molecular surveillance of recently acquired HIV infections in Germany, 2013 to 2014

**DOI:** 10.2807/1560-7917.ES.2017.22.2.30436

**Published:** 2017-01-12

**Authors:** Andrea Hauser, Alexandra Hofmann, Kirsten Hanke, Viviane Bremer, Barbara Bartmeyer, Claudia Kuecherer, Norbert Bannert

**Affiliations:** 1 Division of HIV and Other Retroviruses, Robert Koch Institute, Berlin, Germany; 2 Division of HIV/AIDS, STI and Blood-borne Infections, Robert Koch Institute, Berlin, Germany; 3 Charité – Universitätsmedizin, Berlin, Germany

**Keywords:** Human immunodeficiency virus, HIV drug resistance, HIV subtype, molecular surveillance, recency testing, dried serum spots

## Abstract

To enable an up-to-date molecular analysis of human immunodeficiency virus (HIV) genotypes circulating in Germany we have established a surveillance system based on recently acquired HIV infections. New HIV infections are reported to the Robert Koch Institute as a statutory duty for anonymous notification. In 2013 and 2014, a dried serum spot (DSS) sample was received from 6,371 newly diagnosed HIV-cases; their analysis suggested that 1,797 samples originated from a recent infection. Of these, 809 were successfully genotyped in the *pol* region to identify transmitted drug resistance (TDR) mutations and to determine the HIV-1 subtype. Total TDR was 10.8%, comprising 4.3% with mono-resistance to nucleoside reverse transcriptase inhibitors (NRTIs), 2.6% to non-NRTIs, 3.0% to protease inhibitors and 0.6% and 0.2%, respectively, with dual- and triple-class resistances. HIV-1 subtype B was most prevalent with 77.0%. Non-B infections were identified more often in men and women with heterosexual transmission compared with intravenous drug users or men who have sex with men (79% and 76%, 33%, 12%; all p < 0.05). Non-B subtypes were also more frequently found in patients originating from countries other than Germany (46% vs 14%; p < 0.05) and in patients infected outside of Germany (63% vs 14%; p < 0.05).

## Introduction

Continuous molecular HIV surveillance provides valuable public health information concerning the transmission of drug-resistant viruses and the dynamics of currently circulating variants. Transmitted drug resistance (TDR) has significant clinical consequences as it is associated with an increase in the failure rate of antiretroviral therapy (ART) [1]. With prevalence of TDR (at least one resistance mutation) ranging between 10% and 15% in several European countries and in North America [2-6], the problem is of substantial concern. Genotypic resistance testing is therefore recommended before commencing first-line treatment [7]. The prevalence of TDR in a country is determined by the history of clinical therapy regimens and the failures resulting from resistance. TDR can vary depending on transmission route, country of origin of the patient and country of infection [6]. From a virological perspective, TDR is largely determined by the persistence of the associated mutation(s). Persistence of those mutations depends mainly on the fitness costs to the virus and on the viral genetic background (e.g. chance of compensatory mutations) [8].

Knowledge of currently circulating HIV subtypes in a country and the dynamics of their spread is of epidemiological and clinical relevance. This information affects the safety and accuracy of HIV diagnostics and is valuable for HIV prevention and vaccine development.

The objective of this study was to determine TDR and HIV-1 subtypes in a substantial subset of new HIV-1 infections diagnosed in 2013 and 2014 in Germany. We intended to examine transmissions from a restricted period of time and therefore, only recently acquired infections among the newly diagnosed cases were included. We aimed to analyse TDR and transmitted HIV-1 subtypes in the main transmission groups: men who have sex with men (MSM), persons with heterosexual contact (HET) and people who inject drugs (PWID), considering sex and origin of the infected individual and place of infection. 

## Methods

### Clinical samples

The data protection officer of the RKI and the German Federal Commissioner for Data Protection and Freedom of Information approved the study protocol (III-401/008#0016).

### RNA isolation and HIV sequencing

HIV RNA from 4 x 100 µL DSS was extracted according to the manufacturer’s instructions with the manual NucliSense Magnetic Extraction method for samples collected in 2013 or the automated Biomerieux EasyMag platform for samples collected in 2014 (both bioMerieux, Capronne, France). Viral RNA was quantified using an in house LTR RT-TaqMan PCR according to a modified protocol [9]. As concentration controls, serial dilutions of the IIIB strain of HIV-1 in human HIV-negative plasma was dropped onto filter cards (DPS standard). Samples were analysed using a newly designed HIV-1 group M generic RT-PCR system (in house *pol* RT-PCR) covering the genomic region of HIV-1 protease (AS9–99) and reverse transcriptase (AS1–252) including all resistance-associated positions. The in house *pol* RT-PCR assay consists of two *pol* RT-PCRs yielding two overlapping amplicons: a fragment 1 of 576 bp (primer_A: CCCTCARATCACTCTTTggCARCgA, position 2,252–2,276; Primer_B: CCTAATTgAACYTCCCARAARTCYTgAgT, position 2,799–2,827) and a second fragment 2 of 718 bp (Primer_C: AAACAATggCCATTRACAgARgA, position 2,613–2,636; primer_D: CTAAYTTYTgTATRTCATTgACAgTCCA, position 3,303–3,330). All nucleotide positions refer to sequence of the HXB2 genome (accession number K03455). The detection limit of the in house *pol* RT-PCR was shown to be equivalent to 1,000 copies/mL DPS standard (highest dilution resulting in 100% positive PCR outcome). Population-based sequencing was performed and a consensus sequence from both fragments was obtained.

### HIV-1 subtyping

The HIV-1 subtype was assigned by applying the REGA HIV-1 subtyping tool (REGA HIV Subtyping Tool Version 3.0) and COMET HIV-1 (Version 1.0) [10] to the *pol* sequence. Only subtype classifications based on bootstrap values of > 70% in the tree topology were taken into account. In cases where a subtype or circulating recombinant form (CRF) was not assigned by either subtyping tool, the strain was classified as unique recombinant form (URF). In addition, a distance-based neighbour-joining method and bootstrap (PHYLIP version 3.6) was calculated using an extended panel of subtype reference sequences from the Los Alamos HIV sequence database.

### HIV drug resistance interpretation and calculation of the prevalence of transmitted drug resistance

The World Health Organization (WHO)’s surveillance drug resistance mutations (SDRM) list was used to interpret TDR [11]. Levels of expected resistance to each of the three drug classes nucleoside reverse transcriptase inhibitors (NRTI), non-nucleoside reverse transcriptase inhibitors (NNRTI) and protease inhibitors (PI) as mono, dual or triple class resistance were predicted using the Stanford algorithm (version 7.0). Three levels of resistance were scored: high (R), intermediate (I, intermediate and low), sensitive (S, potentially resistant and sensitive).

### Statistical analysis

Descriptive statistics for continuous variables were calculated as medians and interquartile ranges (IQR). Differences in proportions, odds ratios (OR) and 95% confidence intervals (CI) were assessed by two-sided Fisher’s exact test using EPICALC 2000 software (version 1.02; Gilman and Myatt 1997). A two-sided p value ≤ 0.05 was considered significant.

## Results

### Characterisation of the study population

In 2013 and 2014, a total of 6,371 DSS prepared from residual serum of newly HIV-diagnosed cases were submitted to the RKI along with the anonymous report. Of these, 2,034 (32%) were serologically classified as recent HIV infections using the BED-CEIA. The viral load was reported for 700 and a CD4^+^ T-cell count for 543 of the 2,034 specimens. Specimens with CD4^+^ T-cell counts < 200 cells/µL (n = 108) or viral loads < 400 copies/mL (n = 50) or both (n=8) were reclassified as 'long-standing infections' and, together with repeatedly reported cases (n = 71), excluded, resulting in 1,797 DSS. Sufficient material (four serum spots à 100 µL) was available for 1,387 of those specimens. The *pol* RT-PCR amplification and sequencing was successful in 809 samples; these represented the final study panel of recent HIV infections. A viral load was reported for 298 of 809 cases, with a median of 184,481 copies/mL (IQR: 45,405–983,158). Among these 298 cases, CD4^+^ T-cell counts were available for 214, with a median of 357 cells/µL (IQR: 278–487). The proportional distribution of recently infected cases analysed by sex, transmission routes, country of origin and country of infection extracted from sociodemographic data submitted with the notification form are shown in Table 1.

**Table 1:** Characteristics of patients with recent infection included in molecular HIV surveillance, Germany, 2013–14 (n = 809)

Study population	n	%
**Sex **		
Male	718	88.8
Female	86	10.6
Not reported	5	0.6
**Transmission group**		
Men who have sex with men	497	61.4
Persons with heterosexual contacts	65	8.0
Persons with intravenous drug use	21	2.6
Other	6	0.7
Not reported	220	27.2
**Country of origin**		
Germany	485	60.0
Other	158	19.5
Not reported	166	20.5
**Country of infection**		
Germany	523	64.6
Other	91	11.2
Not reported	195	24.1

### Prevalence of transmitted drug resistance

The overall prevalence of TDR in patients with recently acquired HIV infection diagnosed in 2013 and 2014 was 10.8% (n = 87/809; 95% CI: 8.8–13.1). This comprised mono-resistance to NRTIs in 4.3% (35/809; 95% CI: 3.1–6.0) of the patients, to NNRTIs in 2.6% (21/809; 95% CI: 1.7–4.0) and to PIs in 3.0% (24/809; 95% CI: 2.0–4.5) of cases. Dual and triple class resistance was identified in 0.6% (n = 4 NRTI/NNRTI; n = 1 NNRTI/PI) and 0.2% (n = 2 NRTI/NNRTI/PI) of the patients, respectively (Table 2).

**Table 2:** Prevalence of transmitted drug resistance to the major antiretroviral drug classes in the study population stratified by relevant subgroups, Germany, 2013–14 (n = 809)

	TDR		NRTI		NNRTI		PI		Dual		Multi	
	n	%	n	%	n	%	n	%	n	%	n	%
**Total**	87	10.8	35	4.3	21	2.6	24	3.0	5	0.6	2	0.2
**Sex**												
Male (n = 718)	78	10.9	32	4.5	18	2.5	21	2.9	5	0.7	2	0.3
Female (n = 68)	9	10.5	3	3.5	3	3.5	3	3.5	0		0	
Not reported (n = 5)	0		0		0		0		0		0	
**Transmission group**												
MSM (n = 497)	59	11.9	22	4.4	14	2.8	17	3.4	4	0.8	2	0.4
HET (n = 65)	4	6.2	3	4.6	1	1.5	0		0		0	
PWID (n = 21)	2	9.5	1	4.8	0		1	4.8	0		0	
Other (n = 6)	0		0		0		0		0		0	
Not reported (n = 220)	22	10.0	9	4.1	6	2.7	6	2.7	1	0.5	0	
**Country of origin**												
Germany (n = 485)	56	11.5	24	4.9	12	2.5	16	3.3	3	0.6	1	0.2
Other (n = 158)	12	7.6	2	1.3	5	3.2	3	1.9	1	0.6	1	0.6
Not reported (n = 166)	19	11.4	9	5.4	4	2.4	5	3.0	1	0.6	0	
**Country of infection**												
Germany (n = 523)	63	12.0	23	4.4	15	2.9	18	3.4	5	1.0	2	0.4
Other (n = 91)	5	5.5	3	3.3	1	1.1	1	1.1	0		0	
Not reported (n = 195)	19	9.7	9	4.6	5	2.6	5	2.6	0		0	
**HIV-1** Subtypes												
B (n = 623)	75	12.0	33	5.3	16	2.6	19	3.0	5	0.8	2	0.3
Non-B (n = 186)	12	6.5	2	1.1	5	2.7	5	2.7	0		0	

HET: persons with heterosexual contact; HIV: human immunodeficiency virus; MSM: men who have sex with men; NRTI: nucleotide reverse transcriptase inhibitors; NNRTI: non-nucleotide reverse transcriptase inhibitors; PI: protease inhibitor; PWID: people who inject drugs; TDR: transmitted drug resistance.

`Not reported´ data were excluded from the pairwise comparisons. All p values of pairwise comparison were > 0.05.

**Figure 1:**
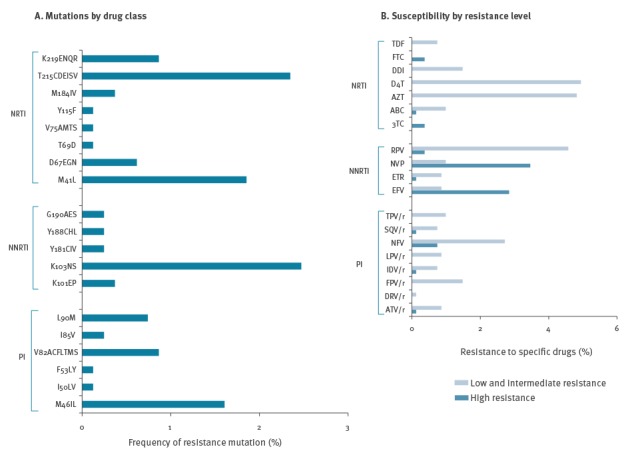
Prevalence of transmitted drug resistance mutations by drug class (A) and predicted susceptibility to antiretroviral drugs by level of resistance (B) in the study population, Germany, 2013–14 (n = 809)

NRTI: nucleoside reverse transcriptase inhibitors; NNRTI: non-nucleoside reverse transcriptase inhibitors; PI: protease inhibitor.

Of all 52 NRTI-associated mutations identified, 46 were thymidine analogue mutations (TAM), namely: the revertants of T215Y (T215CDEISV; 2.3%; 19/809), M41L (2.2%; 15/809), K219ENQR (0.9%; 7/809) and D67EGN (0.6%; 5/809). TAM were selected by the thymidine analogues and conferred intermediate resistance to azidothymidine (AZT) and stavudine (D4T). The most prevalent NNRTI resistance mutations were K103N and K103S (2.5%; 20/809), selected by first generation NNRTIs. The PI-selected resistance mutations M46I and M46L were present at a level of 1.6% (13/809) associated with resistance to a broad spectrum of PIs: atazanavir/ritonavir (ATV/r), fosamprenavir (FPV/r), indinavir (IDV/r), lopinavir (LPV/r), nelfinapir (NFV) and tripranavir (TPV/r). The PI-selected resistance mutations L90M (resistance to LPV/r, ATV/r, saquinavir (SQV)/r, IDV/r, NFV) and V82ACFLTMS (resistance to FPV/r, TPV/r, IDV/r, LPV/r, ATV/r) were present at levels of 0.7% (6/809) and 0.9% (7/809), respectively (Figure 1A and B).

Panel B: Prediction according to Stanford SIR algorithm: intermediate levels of resistance (light colours) and high levels of resistance (dark colours); low and potentially low levels are not indicated.

Drug resistance mutations observed in transmitted HIV strains mainly induced intermediate levels of resistance to NRTI and PI (mainly resistance-associated singleton mutations). High levels of resistance were frequently observed to the NNRTIs efavirenz (EFV) and nevirapine (NVP) and were caused primarily by the prevalent resistance mutation K103N (Figure 1B).

We found no correlation between TDR and sex, transmission group, origin of patient or country of infection in general or for any resistance class (all p values > 0.05) (Table 2).

### Prevalence of HIV-1 subtypes

The most prevalent HIV-1 subtype in the total study population (n = 809) was subtype B with 77.0% (623/809; 95% CI: 73.9–79.8), while 23.0% of HIV-1 infections (186/809; 95% CI: 20.2–26.1) were caused by non-B subtypes including A (5.1%, 41/809), C (3.6%, 29/809), D (0.7%, 6/809), F (1.6%, 13/809), G (2.7%, 22/809), CRF01_AE (2.0%, 16/809) and CRF02_AG (3.3%, 27/809) as well as 4.0% (32/809) other rare CRFs (06_cpx, 07_BC, 12_BF, 18_cpx, 19_cpx, 34_01B, 35_AD, 44_BF, 49_cpx) and URFs. These rare CRFs were grouped together with the URFs into the rare CRF/URF subgroup.

The subtype distribution in the subset of recent infections with available CD4^+^ T-cell counts and viral loads (n = 214) was very similar to the overall study population, with 77.1% (165/214) subtype B and 22.9% (49/214) non-B infections including A (4.2%, 9/214), C (5.1%, 11/214), D (0.9%, 2/214), F (0.9%, 3/214), G (2.8%, 6/214), CRF01_AE (1.9%, 4/214) and CRF02_AG (1.9%, 4/214) as well as 4.7% (10/214) rare CRF/URFs (all p values of pairwise comparisons > 0.2).

HIV-1 subtype B was associated with a significantly higher proportion of TDR (12.0%, 75/623) than the non-B subtypes (6.4%; 12/186; OR: 0.50; 95% CI: 0.27–0.95; p = 0.04), particularly with regard to the NRTI mutations (5.3% in B, 1.1% in non-B, p = 0.01) (Table 2).

**Figure 2:**
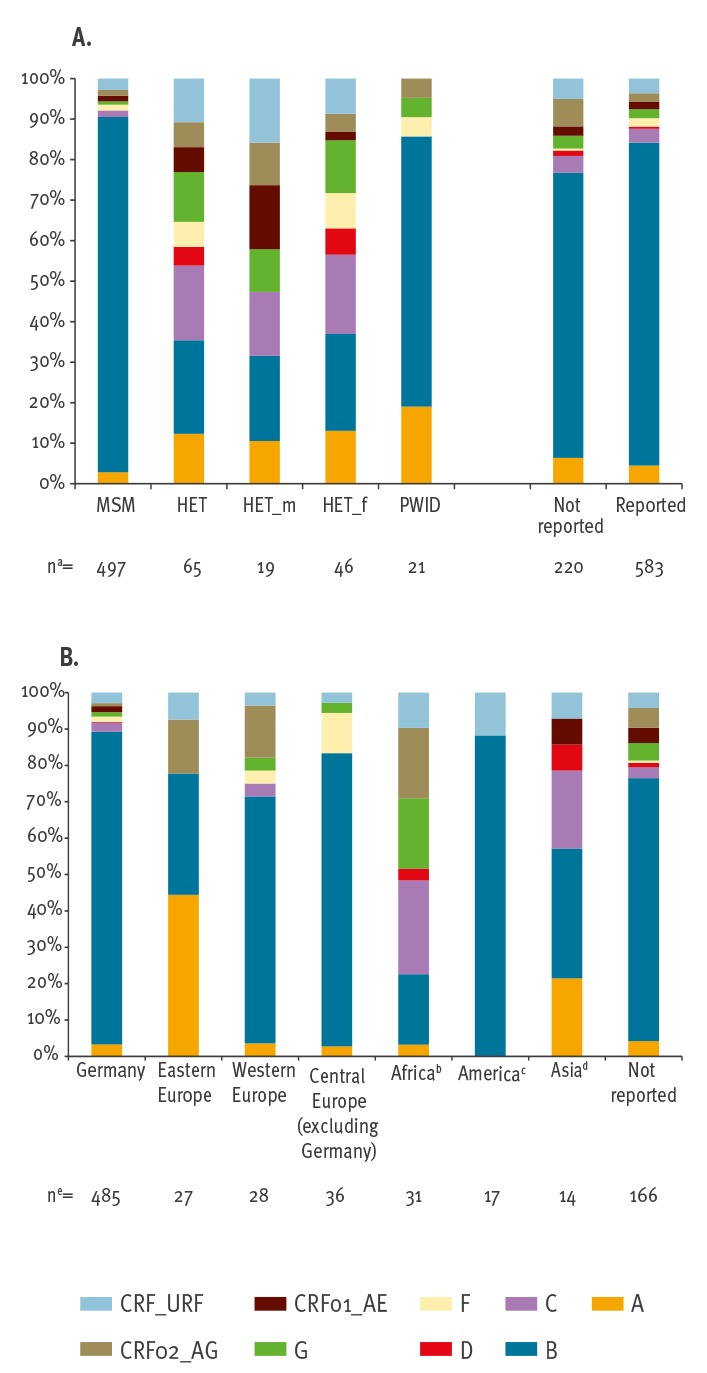
Subtype distribution (A) in the main transmission groups and (B) by country of origin, Germany, 2013–14 (n = 809)

HET: persons with heterosexual contact; HET_f: female persons with heterosexual contact; HET_m: male persons with heterosexual contact; MSM: men who have sex with men; PWID: people who inject drugs.

^a^ Transmission group `other´(n=6, see Table 1) was excluded from the data set.

^b^ Patients from the Middle East, North and sub-Saharan Africa.

^c^ Patients from North and Latin America.

^d^ Patients from South and South-east Asia.

^e^ Two patients from the Caribbean, one patient from Oceania and two patients with unknown non-German origin were excluded.

With regard to the main transmission groups, MSM were predominantly infected by subtype B, with 87.7% (436/497) which is significantly higher than in PWID (14/21) or in HET (15/65; male HET: 4/19 and female HET 11/46; all p values _MSM-PWID-HET_ < 0.05). In HET, subtype diversity was high and particularly non-B subtypes A (8/65), C (12/65) and G (8/65) were frequent. In PWID, subtype A (4/21) was the second most prevalent subtype (after B with 14/21), while subtypes C, D, CRF01_AE and the group of rare CRF/URF were not identified in PWID. Subtype distribution in the group that did not report transmission type mirrored the group that did report transmission type (MSM + HET + PWID), with a slightly lower proportion of subtype B (70.5%, 155/220 vs 79.8%, 465/583) (Figure 2A). The proportion of subtype non-B infections in Germans (14.0%, 68/485) was significantly lower than in individuals from other countries (45.6%, 72/158; p < 0.0001). Subtype B was predominant in Germans and in migrants originating from America and western and central Europe (417/485, 15/17, 19/28 and 29/36, respectively). Subtype A was the most frequently subtype identified in eastern Europeans (12/27), while migrants from Africa and Asia were infected with a greater variety of subtypes (Figure 2B).

**Figure 3:**
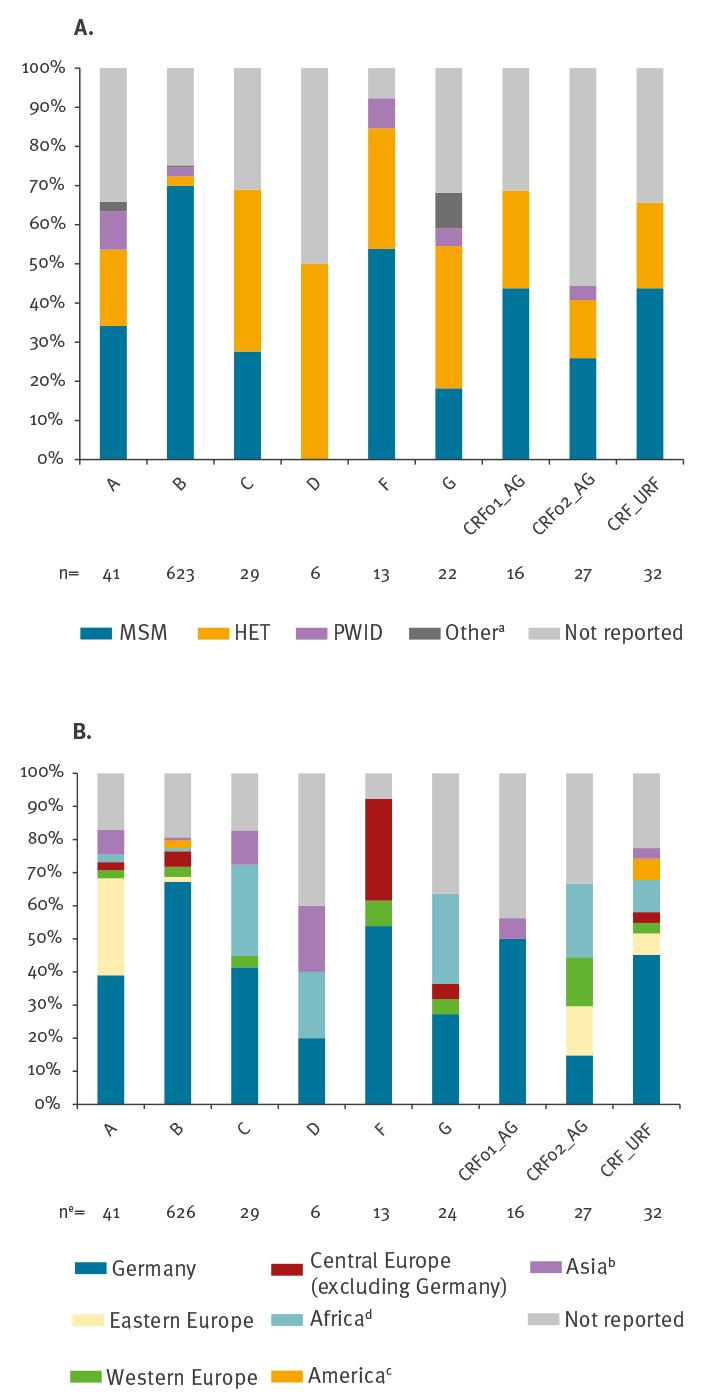
Prevalence of HIV-1 subtypes stratified by (A) main transmission groups and (B) origin of patient, Germany 2013–14 (n = 809)

HET: persons with heterosexual contact; MSM: men who have sex with men; PWID: people who inject drugs.

^a^ Transmission group `other´(n=6), see Table 1.

^b^ Patients from South and South-east Asia.

^c^ Patients from North and Latin America.

^d^ Patients from Middle East, North and sub-Sahara Africa.

^e^ Two patients from the Caribbean, one patient from Oceania and two patients with unknown non-German origin were excluded.

For the subtypes A, B, F, CRF01_AE and the rare CRF/URF, the main transmission route was MSM (14/41, 436/623, 7/13, 7/16 and 14/32, respectively) while the subtypes C, D and G were mainly transmitted by heterosexual contacts (12/29, 3/6 and 8/22, respectively) (Figure 3A).

HIV subtype A was mostly identified in Germans (16/41) and eastern European patients (12/41), subtype C and G in Germans (12/29 and 6/22) and Africans (8/29 and 6/22), while for Subtype D and CRF02_AG, no main origin of patients could be identified. The rare CRF/URFs were mainly identified in Germans (14/31) but also in patients originating from other continents (Europe (4/31), Africa (3/31), America (2/31) and Asia (1/31)) (Figure 3B).

A stratification according to the geographical region of acquisition revealed that in Germany (86.2%; 451/523), America (3/5) and in western (14/21) and central Europe (7/12), subtype B infections were mainly acquired. Non-B subtypes were acquired more frequently abroad (62.6%; 57/91) than in Germany (13.8%; 72/523; p < 0.05).

## Discussion

We have established a molecular HIV-1 surveillance strategy based on samples collected in association with the mandatory notification system of new HIV diagnoses in Germany. Analysis of samples was restricted to recent HIV-transmissions with a defined duration of less than 302 days since HIV infection. This approach permitted us to focus on the viruses circulating during any given period of time and to estimate current trends in the HIV epidemic, because late presenters who acquired an infection before that period were excluded.

The overall TDR prevalence among the 2013 and 2014 study samples was 10.8%. This is largely comparable to previously published cohort studies from Germany and several other western European countries with a long history of combination ART (cART) [3-5] and indicates in many respects that the rate of TDR is essentially stable: Patients included in the German HIV-1 seroconverter cohort between 1996 and 2010 revealed TDR in 11.9% [5]. Two recent comprehensive epidemiological studies involving 26 European countries carried out on behalf of the SPREAD-programme reported levels of 8.9% (95% CI: 8.1–9.8) and 8.3% (95% CI: 7.7–9.5) for the period from 2002 to 2007 [6] and from 2008 to 2010, respectively [12]. Proportions of patients with mono-NRTI, mono-NNRTI, mono-PI, dual- and multi-class resistance (5.0%, 2.9%, 2.5%, 0.8% and 0.4%, respectively) [6] were very similar to our findings (4.3%, 2.6%, 3.0%, 0.6% and 0.2%). In both the SPREAD studies and in ours, the detected NRTI resistance mutations belonged to the TAM (mainly revertants of T215Y, followed by M41L) selected by AZT and D4T. The most prevalent NNRTI SDRMs were K103NS, and resistance to PIs was mainly due to the mutations L90M and M46IL [6,12]. TAM are very frequent and long-term persisting resistance mutations [8]. De novo selection by current therapies is unlikely because the use of AZT and D4T is declining [13]. We therefore suggest that these have become circulating strains by onward transmission from untreated, and probably undiagnosed, individuals. While TAM may not have any particular impact on the success of current first-line treatments, K103N and K103S may still be associated with their failure [7].

Sex was not associated with TDR in our study. However, the prevalence of TDR was slightly but not significantly higher among MSM, in Germans or in individuals infected in Germany. In studies carried out in some other European countries, the associations between TDR and MSM or subtype B (exhibiting higher TDR levels) were significant [6,12,14].

HIV-1 epidemiology is characterised by compartmentalised subepidemics of subtypes with a dynamic nature. The most prevalent subtype in western European countries is subtype B, mainly as a consequence of multiple introductions via migration, tourism and trade from different geographical areas [15]. The lowest proportion of subtype B was reported for Portugal with 48.3% for patients diagnosed between 2006 and 2012 [16] while the highest proportion was observed in Poland with 96.2% for patients diagnosed between 2002 and 2005 [17].

Determinants of subtype distribution are the transmission group, country of origin and country of infection. Subtype B is highly prevalent in MSM, most probably as a result of a founder effect rather than an increased transmission potential of subtype B [18]. Non-B subtypes were significantly more frequently diagnosed in sub-populations originating from and often acquired in countries other than Germany. Most migrants with recent HIV infections carried subtypes dominating in their country of origin [19], suggesting that they may have acquired the infection during a visit to their home countries or within their community in Germany.

Interestingly, a considerable proportion of rare CRF/URFs are present in patients originating from all continents. However, the high proportion of Germans in the non-B clade group, in particular those with subtype A, F and rare CRF/URFs, indicates that non-B strains have become endemic in the German population. Although the number of PWID analysed was low, HIV-1 subtype A was strikingly more common in PWID than in other transmission groups.

One limitation of our study was that the restriction to recently acquired infections over-estimates patient groups that are tested more frequently due to their awareness of transmission risks. This might explain the slightly higher prevalence of MSM among recent infections in our study (61.4%) compared with the notification data for all (recent and prevalent) newly diagnosed HIV infections in 2013 and 2014 (57.7%) [20]. A second limitation was the overall genotyping success rate of 58% in samples from recent infections. This was mainly due to a lack of serum spots for RNA extraction or to RNA degradation after inappropriate handling or shipment. A technical limitation was the expected misclassification of long-standing as recent infections due to a false recent rate (FRR) of ca 4.7% for subtype B or even higher for the non-B subtypes, in particular A and D (FRR 18.9 and 18.2%, respectively), using the BED CEIA assay [21]. This may have led to an over-representation of non-B subtypes in the study. Taking into account CD4^+^ T-cell counts and viral load data as parameters for recency estimation is expected to diminish this bias [22]. Consideration of these clinical parameters that were available for about one third of the samples only marginally reduced the prevalence for the recent non-B infections from 23.0% to 22.9% but, more significantly, from 5.1% to 4.2% for subtype A, which is generally the most strongly affected by BED misclassification. An ancillary analysis of specimens that were reclassified as long-standing infections based on CD4^+^ T-cell counts < 200 cells/µL and viral loads < 400 copies/mL (n = 72) revealed 33.3% non-B and 11.1% subtype A (data not shown), confirming the anticipated over-representation introduced by the BED ELISA. Based on these data, the lack of CD4^+^ T-cell counts and viral loads for two thirds of the sample set is estimated to result in an over-representation of non-B subtypes of less than 2.5%. Being one of two commercially available recency tests, the BED CEIA is used worldwide for epidemiological surveillance studies [23-28], although efforts to evaluate and improve serological recency tests are ongoing [21].

## Conclusion

The TDR prevalence in recent HIV infections among notified newly diagnosed HIV patients in Germany was still high (> 10%) in 2013 and 2014 and was within the range of other European countries, including the proportions of resistance classes. Although the selection for resistant HIV was dramatically reduced by the introduction of cART and new drugs [29], levels of TDR remain stable in all European countries and are still dominated by resistance to NRTI. This is most likely caused by a continued onward transmission of persisting NRTI-resistant strains that emerged as a result of failed treatment regimens during the pre-cART era (1987–96) and not by transmission of resistant viruses from patients failing cART. Therefore, genotypic resistance testing of HIV before first-line treatment needs to be continued. Since therapy-naïve and probably also undiagnosed patients are the predominant source of TDR, early detection of HIV infection followed by early treatment, as recommended in the current guidelines [7], could reduce the transmission of resistant virus [13,30]. Our data also demonstrate that subtype B remains the most frequently transmitted subtype in Germany because of its high prevalence in MSM.
